# Supplementary material for: Exploring Open Dialogue and autism: A qualitative client-perspective study
Source: PLOS Ment Health. 2025 Oct 7;2(10):e0000449. doi: 10.1371/journal.pmen.0000449 (PMC12798624; doi:10.1371/journal.pmen.0000449)
Supplement: Attachment — Submitted filename: Response to reviewers.pdf [file pmen.0000449.s002.pdf]

Dear Reviewer,

Thank you very much for your comments on our manuscript “Exploring Open Dialogue and autism: A qualitative client-perspective study". We highly appreciate the useful comments and suggestions and have revised the manuscript accordingly. Below we provide a point-by-point response to your comments and an explanation on the revisions made to the manuscript.

We look forward to hearing from you regarding our response. If there are any further questions, we will be happy to answer them.

Sincerely,

Karin Lorenz, Msc

Dr. Joyce Bierbooms

Marieke Kneepkens, Msc

Prof. dr. Inge Bongers

----

1. Does this manuscript meet PLOS Mental Health’s publication criteria? Is the manuscript technically sound, and do the data support the conclusions? The manuscript must describe methodologically and ethically rigorous research with conclusions that are appropriately drawn based on the data presented.

Reviewer #1: Yes

Response: We thank the reviewer for this positive evaluation.

2. Has the statistical analysis been performed appropriately and rigorously?

Reviewer #1: N/A

Response: We acknowledge the reviewer’s note.

3. Have the authors made all data underlying the findings in their manuscript fully available (please refer to the Data Availability Statement at the start of the manuscript PDF file)?
The PLOS Data policy requires authors to make all data underlying the findings described in their manuscript fully available without restriction, with rare exception. The data should be provided as part of the manuscript or its supporting information, or deposited to a public repository. For example, in addition to summary statistics, the data points behind means, medians and variance measures should be available. If there are restrictions on publicly sharing data—e.g. participant privacy or use of data from a third party—those must be specified.

Reviewer #1: No

Response: We understand and appreciate the importance of data transparency. However, in this case, we are unable to make the full interview transcripts publicly available due to ethical and privacy concerns. The transcripts contain detailed personal accounts from clients about their experiences in mental health care with the Open Dialogue approach. Because of the specific

content and context, there is a real risk that professionals or others involved in their treatment could recognize them.

Unfortunately, full anonymization cannot be guaranteed, and we feel a strong responsibility to protect the confidentiality and trust of our participants. For that reason, we are not able to share the complete data set publicly. Researchers who are interested in the data are welcome to contact us to discuss possibilities for limited and ethically responsible access.

4. Is the manuscript presented in an intelligible fashion and written in standard English?
PLOS Mental Health does not copyedit accepted manuscripts, so the language in submitted articles must be clear, correct, and unambiguous. Any typographical or grammatical errors should be corrected at revision, so please note any specific errors here.

Reviewer #1: Yes

Response: We thank the reviewer for this positive assessment

5. Review Comments to the Author
Please use the space provided to explain your answers to the questions above. You may also include additional comments for the author, including concerns about dual publication, research ethics, or publication ethics. (Please upload your review as an attachment if it exceeds 20,000 characters)

Reviewer #1: Thank you for the opportunity to review this manuscript. Support services for adults on the autism spectrum is an important area of research that needs more attention. Overall, I found this manuscript a little hard to understand. It would be helpful if the authors could seek advice from an editor to minimise repetition and ensure that the English words used accurately reflect the intended meaning.
Response: Thank you for your valuable feedback. In response, we have carefully reviewed the manuscript, sought advice where necessary, and made the necessary adjustments to reduce repetition and enhance clarity. In the following responses, we address each of your specific points and explain the adjustments we have made accordingly. Line numbers (in parentheses) indicate the corresponding location in the document with visible track changes.

Introduction

- It would be helpful to move the explanation of what Open Dialogue is on Page 4 to the second paragraph in the Introduction. The audience needs to know what Open Dialogue is before reading about its efficacy.
  Response: Thank you for your helpful suggestion. We agree that it makes sense to introduce Open Dialogue earlier in the Introduction. As such, we have moved the explanation of what Open Dialogue is to the second paragraph (Line 57). Additionally, we have relocated the section discussing the efficacy of Open Dialogue below Table 1 (Line 90).

- Reference No. 22 discussed that there is a lack of high quality evidence about Open Dialogue. This should be mentioned in the Introduction.
  Response: We have addressed this point in the Introduction as suggested. To prevent any potential confusion, please note that reference 22 has been renumbered to reference 26 due to changes made in the text.

Line 100: “Despite the growing interest, it has also been noted that there is still a lack of high-quality evidence to fully substantiate the long-term benefits of Open Dialogue [26]. However, several studies have shown that clients perceive the OD approach as distinctive from conventional treatments, […]”.

- P8 Line 112: Are the "promising results from Finland" published or part of a manuscript in preparation? It would be good to include a citation.
  Response: We have added the relevant references [21-24] as suggested, which discuss the promising results from Finland, as previously described in the Introduction.

  Line 137: “Even though the literature does not provide a clear answer, professionals in a specialized mental health practice for autism in the Netherlands were inspired by promising results from Finland [21-24].”

Method

- What does usual care in the MHC involve for clients diagnosed with autism?
  Response: We have added the requested information under section 2.1. Setting.

  Line 163: “Treatment as usual involves regular meetings with a coordinating clinician and a case manager to develop an individualized treatment plan. Outpatient care may include support with daily structure, daytime activities, financial matters, and psychological treatment for comorbid symptoms such as anxiety. Involvement of a client’s social network is possible but not required.”

- Why is the Open Dialogue meeting only offered when the treatment team observes or anticipates a crisis?
  Response: There are two main reasons why OD network meetings were primarily offered in situations where the treatment team observed or anticipated a crisis. First, due to limited capacity — only six professionals within the treatment team were trained in OD — it was not feasible to offer OD to all clients. Second, OD was considered particularly helpful in situations where communication had broken down, trust needed to be rebuilt, or where treatment had reached an impasse. Therefore, the team prioritized offering OD in such high-need contexts, where its potential added value was greatest. We have clarified this in section 2.1. Setting.

  Line 168: “Within the treatment team, six professionals were trained to provide OD care. As a result, it was not feasible to offer OD to all clients, and it was prioritized for situations with the greatest need. These six OD therapists successfully completed the one-year postgraduate training program called “Peer-supported Open Dialogue, Social Network and Relationship Skills” at the Academy of Peer-supported Open Dialogue (APOD) in the United Kingdom, which is accredited by the London South Bank University.
  When the team observed or anticipates a crisis (e.g., when a client was off balance or an impasse had emerged in treatment), or noticed unrest within the client's network, the client was offered the opportunity to participate in an OD network meeting. OD was

considered helpful in restoring communication, rebuilding trust, and creating new possibilities for moving forward."

- Can a client choose to take part in the Open Dialogue meetings if they do not have a crisis?
  Response: Yes, clients could choose to initiate an OD network meeting themselves, even if there was no crisis. However, in practice, most OD meetings were initiated by the treatment team in response to specific needs. We have clarified this in section 2.1. Setting.

  Line 167: "Clients can initiate OD network meetings themselves, but in practice, such meetings are usually initiated by the treatment team."

- The section on recruitment process can be shortened and made clearer.
  Response: We have shortened and clarified the recruitment section to improve readability and focus. The revised version is now more concise while retaining all key details (line 231).

- Although Open Dialogue meetings do not have a pre-defined agenda, it would be good to have some information on what was usually discussed in the Open Dialogue meetings that the participants attended.
  Response: Thank you for the helpful suggestion. We have added the following information to clarify the types of topics typically discussed in the OD network meetings.

  Line 182: "The OD network meetings were held in person, with no predefined agenda. At the beginning of each meeting, attendees were always asked how they wanted to use the meeting time. Examples of topics discussed included challenges in collaboration with professionals, issues arising in relationships between family members, and difficulties in deciding on further treatment options."

- Were Open Dialogue meetings, interviews, and focus groups conducted in person or online?
  Response: The Open Dialogue meetings, interviews, and focus groups were conducted in person. We have made the necessary changes in the manuscript to reflect this.

  Line 182: "The OD network meetings were held in person, […]"
  Line 301: "Both individual interviews and focus groups were conducted in person."

- There is no need to include the names of the themes in the Method section if they are already described in Results.
  Response: Thank you for your suggestion. We have removed the names of the themes from the Method section

- It would be good to add more information on how the research team's background and existing knowledge influenced the analysis process.
  Response: We agree with your suggestion and have added more information about the research team's background in Section 2.3.3. This also allowed us to clarify who was involved in the data collection. Furthermore, in Section 2.4 (Data Analysis), we now describe more explicitly the specific roles of each researcher in the analysis process and how their respective expertise influenced the interpretation of the data.

Line 304: “The research team consisted of four members: two researchers and two supervisors. While all team members had prior knowledge of autism and OD, each contributed their own expertise to the research. One researcher specialized in autism, while the other focused on OD. The supervisors brought extensive experience in qualitative research within the field of mental health care. Interviews were conducted by the researcher with the background in autism, who has extensive experience working with individuals diagnosed with autism and conducting sensitive, in-depth conversations. […]”

Line 353: “During the analysis, the researcher specialized in autism took the lead, as we considered it important that the responses of clients with autism were interpreted appropriately, with an emphasis on inductive analysis. The researcher with expertise in OD was closely involved throughout the entire coding and writing process to ensure accurate interpretation of the OD approach, with a more deductive focus. Code generation and theme development were discussed extensively to reach consensus. The supervisors were involved at predetermined intervals and were also consulted by the researchers whenever uncertainties arose.”

- It's a bit unclear how many researchers were involved in each stage of the analysis process, as the plural "researchers" is used throughout but P15 mentions a second researcher.
  Response: We understand the confusion and hope that the revised description, as outlined in our response above, has clarified this point as well. Specifically, we replaced the sentence “A second researcher was involved throughout the entire coding (and writing) process, and both codes and themes were subjected to discussion to achieve consensus.” with a more detailed explanation of the roles and involvement of each team member during the analysis. This revision clarifies which researchers were involved at each stage and how their expertise shaped the analytical process.

  Line 353: “During the analysis, the researcher specialized in autism took the lead, as we considered it important that the responses of clients with autism were interpreted appropriately, with an emphasis on inductive analysis. The researcher with expertise in Open Dialogue was closely involved throughout the entire coding and writing process to ensure accurate interpretation of the OD approach, with a more deductive focus. Code generation and theme development were discussed extensively to reach consensus. The supervisors were involved at predetermined intervals and were also consulted by the researchers whenever uncertainties arose.”

Results

- It would be helpful to reduce the number of quotes and use them selectively to illustrate ideas.
  Response: We have reviewed the quotes in the Results section and have removed several, while selectively retaining those that best illustrate key ideas. A few quotes have also been shortened to ensure clarity and relevance.

Discussion

- Some parts of the Discussion feel a bit repetitive. It would be helpful to re-organise this section to ensure each idea is discussed once.
  Response: Thank you for this valuable feedback. Upon revisiting the Discussion section in light of your feedback, we acknowledge the repetition that was present in the original version of the Discussion section. In response, we have substantially revised and reorganized the Discussion to improve clarity and flow, ensuring that each idea is addressed only once and in a more structured manner.

- It would be helpful to suggest how the Open Dialogue approach might be adapted to make it more comfortable for people who have trouble managing uncertainty.
  Response: Thank you for this helpful suggestion. We have incorporated it into the latter part of the Discussion, where we reflect on how the Open Dialogue approach might be adapted to better support individuals who experience difficulties with managing uncertainty.

  Line 692: "Reflecting on practical implications, it may be beneficial to consider adaptations to the Open Dialogue (OD) approach to make it more comfortable for individuals who experience difficulties with managing uncertainty. One suggestion that emerged from the data was to better prepare participants by providing a clearer picture of what to expect during the network meeting, as well as encouraging them to reflect in advance on topics they may wish to discuss. Such preparatory steps could enhance participants' sense of agency and potentially support the recovery process. Similar recommendations regarding the value of preparatory guidance have been made in previous research [30]."

6. PLOS authors have the option to publish the peer review history of their article (what does this mean?). If published, this will include your full peer review and any attached files.
Do you want your identity to be public for this peer review? If you choose "no", your identity will remain anonymous but your review may still be made public.
For information about this choice, including consent withdrawal, please see our Privacy Policy.

Reviewer #1: No

Response: We acknowledge the reviewer's choice to remain anonymous.
