# Supplementary material for: Exploring Open Dialogue and autism: A qualitative client-perspective study
Source: PLOS Ment Health. 2025 Oct 7;2(10):e0000449. doi: 10.1371/journal.pmen.0000449 (PMC12798624; doi:10.1371/journal.pmen.0000449)
Supplement: Attachment — Submitted filename: Response to Reviewers 2.0.pdf [file pmen.0000449.s003.pdf]

Dear Reviewer and editor,

Thank you very much for your comments on our manuscript “Exploring Open Dialogue and autism: A qualitative client-perspective study". We understand the need for one more reviewer.
We highly appreciate the useful comments and suggestions and have revised the manuscript accordingly. Below we provide a point-by-point response to the comments and an explanation on the revisions made to the manuscript.

We would also like to clarify that content of this manuscript has been included as a chapter in the doctoral dissertation of the first author, which soon will be available through the university repository. The dissertation is not commercially published and does not have an ISBN. The version in the dissertation reflects the manuscript as it was originally submitted to *PLOS Mental Health* on October 15, 2024. We trust this will not pose any issues for the review and potential publication process, but we wanted to be transparent about this in case clarification was needed.

Thank you for your willingness to assess the revised manuscript in-house to save time. We look forward to hearing from you regarding our response. If there are any further questions, we will be happy to answer them.

Sincerely,

Dr. Karin Lorenz

Dr. Joyce Bierbooms

Marieke Kneepkens, Msc

Prof. dr. Inge Bongers

---

1. If the authors have adequately addressed your comments raised in a previous round of review and you feel that this manuscript is now acceptable for publication, you may indicate that here to bypass the “Comments to the Author” section, enter your conflict of interest statement in the “Confidential to Editor” section, and submit your "Accept" recommendation.

Reviewer #1: All comments have been addressed

Reviewer #2: (No Response)

---

2. Does this manuscript meet PLOS Mental Health’s publication criteria? Is the manuscript technically sound, and do the data support the conclusions? The manuscript must describe methodologically and ethically rigorous research with conclusions that are appropriately drawn based on the data presented.

Reviewer #1: Yes

Reviewer #2: Yes

---

3. Has the statistical analysis been performed appropriately and rigorously?

Reviewer #1: N/A

Reviewer #2: N/A

---

4. Have the authors made all data underlying the findings in their manuscript fully available (please refer to the Data Availability Statement at the start of the manuscript PDF file)?

The PLOS Data policy requires authors to make all data underlying the findings described in their manuscript fully available without restriction, with rare exception. The data should be provided as part of the manuscript or its supporting information, or deposited to a public repository. For example, in addition to summary statistics, the data points behind means, medians and variance measures should be available. If there are restrictions on publicly sharing data—e.g. participant privacy or use of data from a third party—those must be specified.

Reviewer #1: No

Reviewer #2: No

Response: We understand and appreciate the importance of data transparency. However, in this case, we are unable to make the full interview transcripts publicly available due to ethical and privacy concerns. The transcripts contain detailed personal accounts from clients about their experiences in mental health care with the Open Dialogue approach. Because of the specific content and context, there is a real risk that professionals or others involved in their treatment could recognize them.

Unfortunately, full anonymization cannot be guaranteed, and we feel a strong responsibility to protect the confidentiality and trust of our participants. For that reason, we are not able to share the complete data set publicly. Researchers who are interested in the data are welcome to contact us to discuss possibilities for limited and ethically responsible access.

---

5. Is the manuscript presented in an intelligible fashion and written in standard English?

PLOS Mental Health does not copyedit accepted manuscripts, so the language in submitted articles must be clear, correct, and unambiguous. Any typographical or grammatical errors should be corrected at revision, so please note any specific errors here.

Reviewer #1: Yes

Reviewer #2: Yes

---

6. Review Comments to the Author

Please use the space provided to explain your answers to the questions above. You may also include additional comments for the author, including concerns about dual publication, research ethics, or publication ethics. (Please upload your review as an attachment if it exceeds 20,000 characters)

Reviewer #1: (No Response)

Reviewer #2: This paper by Lorenz-Artz and colleagues provides a qualitative exploration of the Open Dialogue method is an autism care setting. The authors utilise focus group discussions and individual interviews to explore client perspectives of this model of care. It is unique in that it gives voice to autistic individuals who historically are often not directly heard from in research.

ABSTRACT
Satisfactory.

INTRODUCTION
Satisfactory as revised.

METHODS

- It might be helpful to further clarify how clients for OD are selected. Were there explicit criteria or it is left to individual judgement? There is some content in this regard in lines 152-155 – perhaps the authors might want to bring that up to align with lines 146-148.
  Response: Thank you for this helpful suggestion. The criteria applied by the team were indeed described in lines 152–155. These passages have now been moved upward to align with the section in lines 146–148, as you suggested. In addition, we clarified that while the treatment team often initiated the idea of offering an OD network meeting - typically when a crisis was observed or anticipated (e.g., when a client was off balance, an impasse had emerged in treatment, or unrest was noticed within the client's network) - an individual professional within the team could also propose this option. Ultimately, there were no fixed, formalized criteria.

  We revised the manuscript accordingly (see lines 146-151 in the 'Revised Manuscript with Track Changes'): *"Clients can initiate OD network meetings themselves, but in practice, such meetings are usually initiated by the treatment team. In principle, all clients under the care of this outpatient team are eligible for Open Dialogue. When the team or an individual professional observed or anticipated a crisis (e.g., when a client was off balance or an impasse had emerged in treatment), or noticed unrest within the client's network, the client was offered the opportunity to participate in an OD network meeting. OD was considered helpful in restoring communication, rebuilding trust, and creating new possibilities for moving forward."*

- *Line 167: for 'gather' read 'gathering'*
  Response: In a revision addressing the feedback point on the study design below, this part of the text was adjusted, and the term 'gather' no longer appears. Therefore, this specific comment is no longer applicable.

- Study Design: the reviewer wonders if the investigators would like to explicitly situate their study within standard qualitative research method. 1) Triangulation – this is the practice of utilising more than one approach (as done in this paper) in order to arrive at more robust findings. It might have been desirable, albeit noting the constraints of willing participants, to have different individuals participate in the FGD and individual interviews. 2) Saturation – this is the practice of gathering qualitative data until additional units do not confer

incremental information. Although mention is made of this under the discussion, the means by which this is said to have been achieved is rather unconvincing. 3) How were the focus groups moderated?

Response: We thank the reviewer for these helpful points. We have revised the 2.2. Design section to explicitly situate the study within qualitative methodology and to clarify triangulation, saturation, and moderation procedures:

- Triangulation: We used methodological triangulation by combining focus groups and semi-structured interviews to generate both interactive, collectively negotiated accounts and confidential, in-depth personal narratives. To avoid carry-over effects and to strengthen independence between datasets, participants were assigned to one modality only: individuals took part either in a focus group or in an interview; no participant took part in both. We have now stated this explicitly.
- Saturation: We agree that a clear account of how saturation was assessed is necessary. In practice, data collection and analysis proceeded iteratively, and we monitored the extent to which new interviews and focus groups generated novel codes or themes. Toward the end of the study, we observed that no substantial new insights were emerging, which indicated that saturation had been reached. At the same time, the study timeline was constrained because one of the investigators could no longer allocate additional time to data collection. Had we not observed saturation, we would have sought to re-approach participants who had been unable to attend the second planned focus group and invite them for interviews. Thus, while practical considerations contributed to stopping recruitment, our judgment that saturation had been achieved was based on the analytic process. We expanded our description of how saturation was assessed.
- Moderation: We now describe the focus-group procedures in more detail. Each focus group was moderated by a member of the research team with a background in autism and extensive experience in working with individuals diagnosed with autism and conducting sensitive, in-depth conversations. Importantly, this moderator was not involved in the care of any participant. She was supported by a second researcher who was trained in the OD approach and experienced in conducting in-depth interviews. This second researcher managed logistics, took observational notes, and, where appropriate, contributed to the discussion to deepen exploration of participants' perspectives. The moderator followed a semi-structured topic guide, encouraged balanced participation, and emphasized confidentiality and psychological safety throughout.

These changes are reflected in the revised manuscript.

Methods, lines 171–220 in the 'Revised Manuscript with Track Changes':
" In this exploratory study, we employed a qualitative multi-method design that combined semi-structured individual interviews and focus groups [46]. The two methods were used for methodological triangulation: focus groups elicited interactional sense-making and participants' experiences with the OD approach, while interviews enabled participants to discuss sensitive personal experiences in a confidential setting [47]. To maintain independence between datasets and prevent carry-over, participants were allocated to one modality only (focus group or interview). […]

Data collection and analysis proceeded iteratively, using a hybrid thematic analysis that combined deductive and inductive approaches [48]. This strategy leverages the strengths of both techniques to provide a more comprehensive grasp of the data [49]. Deductive coding was guided by key concepts from the OD literature, while inductive coding allowed themes to emerge from participants' accounts of their experiences with OD in the context of an autism diagnosis. Given the existing literature on OD, but the limited knowledge of how clients diagnosed with autism experience this approach, it was important to apply both analysis approaches.
Saturation was assessed pragmatically by monitoring whether additional interviews or focus groups generated new codes or themes relevant to the research questions. Toward the end of the study, no substantial new insights were identified, indicating that saturation had been achieved. At the same time, data collection was constrained by the limited availability of one of the researchers. Had saturation not been observed, the OD-trained researcher would have re-approached participants who had been unable to attend the second planned focus group and invited them to take part in individual interviews. Thus, while practical considerations also contributed to ending recruitment, our conclusion that saturation had been reached was ultimately based on the analytic process.

Each focus group was moderated by a member of the research team with a background in autism and extensive experience working with individuals diagnosed with autism and conducting sensitive, in-depth conversations. Importantly, this moderator was not involved in the care of any participant. She was supported by a second researcher who was trained in the OD approach and experienced in qualitative interviewing. This second researcher managed logistics, took observational notes, and, where appropriate, contributed to the discussion to deepen the exploration of participants' perspectives. The moderator followed a semi-structured topic guide, encouraged balanced participation, and emphasized confidentiality and psychological safety throughout."

Discussion, lines 588-590 in the 'Revised Manuscript with Track Changes':
"[…] As described in the Study Design section, data saturation was assessed within the research site and was indicated by the observation that no new aspects emerged in the final focus group. In addition, the anonymized and aggregated results, as described in the results section, were presented to the mental healthcare team at the studied site, and they affirmed the consistency between our findings and their own experiences, providing no additional feedback. […]"

- Line 201: delete 'an'
  Response: Corrected as suggested (line 243 in the 'Revised Manuscript with Track Changes').

- There appears to be an inconsistency between the study design and the details provided under recruitment (lines 214-222). Perhaps the authors might want to include and align the additional detail under study design.
  Response: We thank the reviewer for pointing out this inconsistency. The Study Design section originally described our intended sequence of focus groups followed by interviews, but in practice, recruitment challenges required some flexibility. To clarify, while the initial

plan was to start with focus groups and subsequently conduct interviews, participant availability and timing constraints led us to invite early applicants for individual interviews first, with later participants forming focus groups. This revised description aligns the study design with the recruitment process and accurately reflects the sequence in which data were collected.

We revised the manuscript accordingly (see lines 180-185 in the 'Revised Manuscript with Track Changes'):
"The initial plan was to conduct focus groups first, followed by individual interviews to explore specific aspects in more depth. In practice, recruitment constraints required early applicants to be invited for interviews first, while later applicants participated in focus groups. Despite this change in sequence, the combination of interviews and focus groups ensured comprehensive coverage of the research questions, and no inconsistencies were observed in the data."

- To what extent might the responders on interview have been influenced by prior participation in focus groups?
  Response: We acknowledge and appreciate the reviewer's comment on this matter. To maintain independence between datasets and prevent carry-over effects, participants were explicitly assigned to one modality only: they took part either in a focus group or in an individual interview, but not both. While some participants were absent from focus groups and could later be invited for interviews, no participant took part in both a focus group and an interview. This procedure, now clarified in the manuscript, ensured that interview responses were not influenced by prior participation in focus groups.

  Design, lines 175-177 in the 'Revised Manuscript with Track Changes':
  "To maintain independence between datasets and prevent carry-over, participants were allocated to one modality only (focus group or interview)."

  Recruitment, lines 290-294 in the 'Revised Manuscript with Track Changes':
  "In the first focus group, two participants were absent, one for an unknown reason and the other due to last-minute COVID-19 concerns. In the second focus group, four out of six participants were absent. Due to logistical and time constraints associated with study requirements of one of the involved researchers, we could only approach the absent participants from the first group after its completion. Importantly, participants were assigned to only one data collection modality: those who did not participate in a focus group could later be invited to an individual interview, but no participant took part in both a focus group and an interview. This ensured that the datasets remained independent and that interview responses were not influenced by prior participation in focus groups."

RESULTS
Satisfactory as revised.

DISCUSSION

- Given the cognitive profile of study participants, who were of average to high cognitive level and with good verbal competence, it might be worth discussing how well this model of care is suited to other autistic individuals.
  This also applies to the theme of uncertainty in relation to the autism phenotype. Is there a possibility that the challenges with uncertainty might be related to key features of the autism phenotype?
  Response: We thank the reviewer for drawing attention to this point. As noted, our participants were of average to high cognitive level and possessed good verbal competence. We acknowledge that this may limit the generalizability of our findings to the broader population of individuals diagnosed with autism, particularly those with lower cognitive or verbal abilities. We also agree that challenges with uncertainty may be closely related to key features of the autism phenotype. In the revised discussion, we explicitly address this issue, noting that the experiences reported in this study primarily reflect cognitively able and verbally competent participants, and that further research is needed to explore the applicability of the OD approach across a broader spectrum of individuals diagnosed with autism.

  We have added the following paragraph to the discussion section of the manuscript and revised the Conclusion and future research section (lines 655-661 in the 'Revised Manuscript with Track Changes').

  Discussion, lines 577-589 in the 'Revised Manuscript with Track Changes':
  "Although this study shows promising results regarding the suitability of OD for individuals diagnosed with autism, it is important to consider that many autistic individuals experience distress even with slight uncertainty, which can exacerbate difficulties with social interaction [74-76]. Tolerance of uncertainty is also known to vary with features of the autism phenotype, with severity of ASD predicting levels of intolerance [77,78]. Interventions that rely heavily on verbal expression in conversation are inherently social processes, requiring building rapport, engaging in personal disclosure, and navigating complex social interactions [79-81]. Executive functioning difficulties, social communication challenges, and high levels of alexithymia further complicate engagement in such therapeutic interventions [82, 83]. These considerations highlight that the positive experiences reported here may not fully generalize to individuals with lower cognitive or verbal abilities or those with more severe ASD traits."

  Conclusion and future research, lines 652-660 in the 'Revised Manuscript with Track Changes':
  "Our results suggest that OD care can be offered to individuals diagnosed with autism, although this should be stated with caution due to the small sample size and study design limitations. At the same time, it should be noted that participants in this study were of average to high cognitive level and possessed good verbal competence. Consequently, the experiences reported here may not fully generalize to individuals with lower cognitive or verbal abilities or higher severity of ASD traits, for whom OD may not be the most appropriate form of care. Future research should further explore this promising avenue, examining the applicability, effectiveness, and nuances of the OD approach within a broader spectrum of the population diagnosed with autism."

- It would have been good to have more discussion of non-responders, and whether their reasons for not participating may represent shared features with the seeming minority of responders who had negative views of OD. Given a response rate of approximately 33 per cent, the attitudes of the majority who constitute non-responders might have a lot to say about the larger autism community.
  Response: Thank you to the reviewer for pointing out this important aspect. We acknowledge that the study's response rate of approximately 33% means that the majority of potential participants did not take part, and their perspectives may indeed differ from those reported here. It is possible that some non-responders might share features with the minority of participants who expressed negative views of OD, such as difficulties with uncertainty or discomfort in group discussions. At the same time, the study findings were discussed at an aggregated level with the treatment team, who recognized and supported the reported results. While it remains possible that the attitudes of non-responders could alter the proportion of positive and negative experiences, the continued engagement and enthusiasm of the team in offering OD suggests that a large majority of clients were likely to have experiences consistent with the positive outcomes reported. Nonetheless, we agree that it is important to acknowledge non-responders in the article, and we have added the following statement to the discussion.

  We revised the Limitation section of the manuscript accordingly (see lines 619-630 in the 'Revised Manuscript with Track Changes'):
  "It should be noted that the study's response rate was approximately 33%, meaning that the majority of potential participants did not take part. The perspectives of non-responders may differ from those reported here, and some may have shared features with the minority of participants who expressed negative views of OD. However, the anonymized and aggregated results were presented to the treatment team, who affirmed their consistency with the team's broader experiences, providing no additional feedback. This suggests that the findings reasonably reflect the client group within the studied setting. Still, given the heterogeneous nature of autism [43], generalization to the wider autism population should be made with caution, and further research is warranted."

---

7. PLOS authors have the option to publish the peer review history of their article (what does this mean?). If published, this will include your full peer review and any attached files.

Do you want your identity to be public for this peer review? If you choose "no", your identity will remain anonymous but your review may still be made public.

For information about this choice, including consent withdrawal, please see our Privacy Policy.

Reviewer #1: No

Reviewer #2: No
